# The Establishment of Artificial RNA Cascade Circuits for Gene Regulation Based on Doxycycline-Induced Pre-mRNA Alternative Splicing

**DOI:** 10.3390/ijms26031163

**Published:** 2025-01-29

**Authors:** Guimin Dai, Jiawen Cheng, Weiran Liu, Xueli Yin, Yuanyuan Zhang

**Affiliations:** 1 School of Life Science, Anhui Medical University, Hefei 230032, China; ddaiguimin@163.com (G.D.); cjw001022@163.com (J.C.); wrliu0815@163.com (W.L.); 2 School of Basic Medical Sciences, Anhui Medical University, Hefei 230032, China

**Keywords:** artificial, RNA cascade circuit, alternative splicing, gene regulation

## Abstract

This study developed an artificial chimeric intron module with an RNA riboswitch and TetR aptamer that were integrated into essential gene exons. Doxycycline can modulate Pre-mRNA alternative splicing, modify the exon reading frame, and dynamically regulate gene expression. By shifting the aptamer 2 base pair within the switch, we unexpectedly obtained the “on-switch” CTM and “off-switch” C2ITetR>4A, which possess thoroughly contrasting regulatory functions. The CTM module can conditionally induce tumor cell apoptosis and regulate genes reversibly and sustainably following doxycycline induction. We integrated the C2ITetR>4A/CTM switches with the L7Ae/k-turn module to create an intron-spliced double-switched RNA cascade system. The system can both activate and inhibit the splicing mechanism utilizing the same ligand to minimize crosstalk among aptamer switching elements, control target gene leakage, and enhance the dynamic range of gene expression. We analyzed numerous factors affecting Pre-mRNA splicing to identify the optimal equilibrium point for switch regulation. This will enable precise predictions of dynamic regulatory efficiency and the rational design of genetic modules, thereby providing a valuable instrument for mammalian synthetic biology.

## 1. Introduction

Alternative splicing (AS) of pre-mRNA is an essential step in post-transcriptional mRNA processing. Since Gilbert [1] first established the notion of AS in 1978, numerous studies have shown the crucial role it plays in higher biological systems [2,3]. The failure of splicing regulation may result in multiple genetic disorders [4,5].

Among the few therapeutic strategies presently accessible for conditions resulting from splicing mutations, non-coding RNAs (ncRNAs), as well as antisense oligonucleotides (ASOs), necessitate comprehensive screening and structural alterations [6,7]. Moreover, viral vector insertion modification and CRISPR gene editing entail the risk of ambiguous therapeutic targeting [8,9]. 

Now, reprogramming aberrant splicing presents an innovative strategy for gene therapy targeting-associated diseases [10,11,12,13,14,15]. The development of synthetic biology, especially via the design, alteration, reconstruction, or synthesis of biological components, has yielded novel solutions across various domains. Notably, RNA switches, as “engineered RNA elements”, have the ability to modify their structures upon binding to ligands and thus control gene expression in a spatial, temporal, and dose-dependent manner [16,17,18,19,20,21]. The adaptable architecture of RNA switches facilitates an effortless incorporation with pre-mRNA. Thus, the integration of diverse aptamers with RNA switches can be extensively utilized across multiple cellular systems, ranging from *E. coli* to humans, facilitating the multi-tiered regulation of gene expression [22,23].

Splicing regulation by RNA-based systems has been demonstrated in yeast, plants, elegans, and mammalian cells [24,25,26,27,28,29]. Although its regulatory efficiency can attain up to a 16-fold enhancement in yeast, it generally achieves only a two- to four-fold enhancement in mammalian cells. This disparity constitutes a substantial obstacle to the practical implementation of this method in gene therapy. Consequently, improving regulatory efficiency has emerged as a primary area of the research. This necessitates focusing on two primary elements: (1) augmenting the splicing efficiency at the splice sites and (2) improving the ON/OFF ratio of the switching system in the reaction to ligand molecules [30].

The process of pre-mRNA splicing is intricate, with numerous factors influencing splicing efficiency. The studies indicate that RNA motifs play a crucial role in the selection of splicing sites [31,32]. Additionally, the modification of RNA switches secondary structures and the dynamic regulation of ligand molecules inherently associated with these motifs [33,34,35]. The computational tools, including Human Splicing Finder (HSF) [36], MaxEnt [37], Hbond score (HBS) [38], and GeneSplice [39], can predict the correlation between pre-mRNA motifs and splicing efficiency from various angles. Nevertheless, there are limited instances offering comprehensive experimental evidence regarding the impact of RNA conformation alterations on splicing sites. Thus, we employed MFold’s thermodynamic analysis of RNA secondary structure to examine the influence of structural alterations on splicing-site accessibility. We sought to identify the optimal equilibrium for the dynamic regulation of RNA switch by integrating various factors that influence pre-mRNA splicing, which will serve as an essential instrument for precisely forecasting regulatory efficacy and systematically constructing genetic modules.

In the initial experiment of our research, by utilizing the experimental methodology of the Suess group [29], we construct a chimeric intron module, “CTM”, which contains an RNA riboswitch and TetR aptamer. This type of riboswitch, however, can only be activated by small molecules or a one-way “on-type” regulation. Due to the operation of the artificial switch depending on the conformation alterations in RNA, there is no strict blocking effect on splicing factors. As a result, it still suffers from issues, such as a leaky basal expression, making it difficult to achieve the desired regulatory efficiency.

Fortunately, by moving the position of the TetR aptamer within the switch, we unexpectedly discovered the activation of the cryptic 5’ splice site downstream, which allowed us to adjust the function of splicing regulation elements (SREs). Consequently, we successfully obtained a “C2ITetR” structure, which exhibits an opposing regulatory effect on the CTM.

This “off-type” switch broadens our range of control strategies. Using the principle that ribosomal protein L7Ae from *Staphylococcus xanthobacterium* [40,41,42,43] binds to the K-turn RNA motif, we incorporated these components into the C2ITetR>4A and CTM systems to create a “turn-on” type of combined gene circuit. This intron splicing-based double-switch RNA cascade system improves the control of target gene expression by simultaneously activating and inhibiting the Dox-induced splicing system, thereby exhibiting a high dynamic range and low basal activity. Its ON/OFF activation ratio regulating mCherry gene expression in HeLa cells increased from 3.6-fold to 7.3-fold compared to a single “on-type” switch, demonstrating a superior performance compared to the existing mammalian switches.

Additionally, we show that translational activation may be regulated in a reversible manner by precisely adjusting the drug concentration, thereby enabling an effective control over cell fate via RNA delivery alone. Furthermore, we maintain sequences proximate to splicing sites in order to ensure the accurate identification of the 5’SS, which creates a durable genetic device for controlling the expression of any gene located downstream from the start code. We regulated herpes simplex virus 1 thymidine kinase (HSV-TK) by the CTM switch system and demonstrated target cell-specific apoptosis induction that is dependent on both target Dox and ganciclovir (GCV). This method offers a valuable tool for mammalian synthetic biology research and shows great potential to address diseases associated with abnormal splicing by facilitating the design of cell-type-specific and conditionally triggered RNA circuits.

## 2. Results 

### 2.1. Dox-Dependent “Switch-On” Device 

Intron retention is a form of alternative splicing in which one or more introns remain unspliced in mRNA containing several exons [44]. The aim of our research was to strategically place the “RNA riboswitch” near the 5’ splice site (5’SS) of the intron, in order to regulate RNA conformation with small-molecule drugs, thereby precisely controlling intron splicing and gene expression.

**Figure 1:**
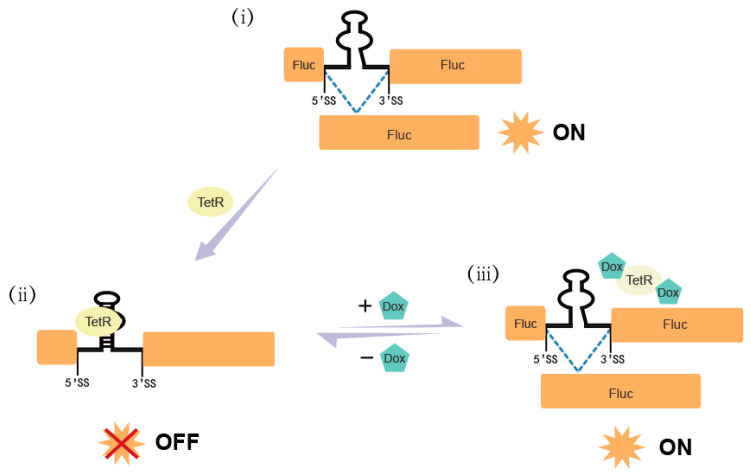
Schematic diagram of Fluc gene regulation via alternative splicing using a Dox-dependent “switch-on” device.

The chosen intron sequence comprises a 5’SS derived from human beta-globulin and a 3’ splice site (3’SS), both exhibiting favorable splicing properties [27]. We created a chimeric intron by introducing a fusion construct comprising an RNA riboswitch and a TetR aptamer into this particular intronic region in order to confer an engineered regulatory function. As shown in Figure 1, this integrated device is located in the Fluc gene, allowing for the reversible modulation of Fluc expression through interactions between the TetR aptamer and its corresponding ligand: without doxycycline (Dox), the aptamer’s binding to the TetR protein forms a stem–loop structure. This distorts the initial assembly of the 5’SS spliceosome, retaining the introns and resulting in an incorrect reading frame for the Fluc gene. However, Dox enables TetR to be released from aptamer RNA, exposing the original 5’SS and allowing intron removal to restore normal gene expression.

#### 2.1.1. Construction and Optimization of the Switch-On Device “CITetR”

The intron device is inserted into the Fluc exon and transferred to cells that consistently express the TetR protein, reflecting the structure’s splicing efficiency via Fluc expression. According to the previous research, many factors influence splicing accuracy and efficiency, particularly the short sequence of the exon–intron boundary near the 5’SS [45,46,47], splicing regulatory elements (SREs), and the short sequence near the 3’ SS, which have a significant effect on splicing efficiency. Thus, we focused on optimizing these key regions to improve the regulatory efficiency of this module by introducing mutations in a number of related sequences.

**Figure 2:**
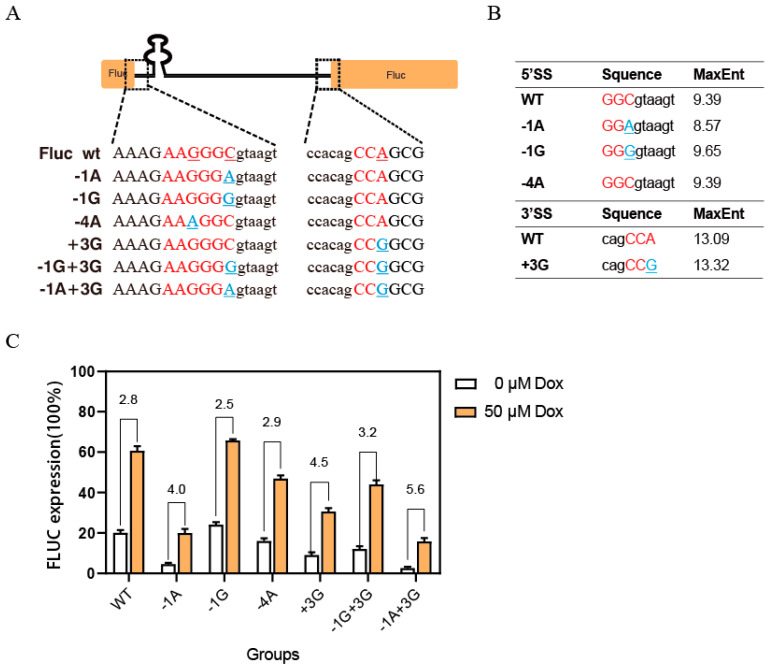
(**A**) Schematic diagram of artificial chimeric introns (CITetRs) inserted into Fluc and mutation sites. Uppercase letters: exons; lowercase letters: introns; red: wild-type sequence; blue: point mutation; “−”: the upstream of 5’SS; “+”: the downstream of 3’SS. (**B**) MaxEnt prediction scores for mutants at the 5’SS and 3’SS. (**C**) The expression of Fluc in various mutant constructs with Dox was regulated. The error bars represent the mean ± s. e. m.

Firstly, the effect of mutations on the exon–intron boundary sequence was examined. Specifically, a series of mutations was introduced into the Fluc exon ranging from −6 bp to −1 bp, upstream of the 5’SS of the aforementioned intron (Figure 2A). MaxEnt, a framework for modeling sequence motifs based on the maximum entropy principle (MEP), was used to predict the RNA splicing signals in short sequences. It was predicted that the −1C>G(−1G) mutation would exhibit a higher splicing efficiency than the wild type (9.65 vs. 9.39). This enhanced splicing capacity was further confirmed by higher luciferase expression levels. In contrast, the −1C>A(−1A) mutation significantly reduced the efficiency of spliceosome recognition, as evidenced by a lower splicing score (8.57) (Figure 2B,C).

We further investigated the impact of SREs. We found that the mutation −4G>A (−4A) reduced luciferase expression with a higher splicing score. Therefore, a highly effective splicing silencer (ESS) binding matrix (sequence: AAAGGC) near the splicing site that specifically interacts with hnRNPA1 (heterogeneous nuclear ribonucleoprotein A1) was discovered when we used the online bioinformatics tool, Human Splicing regulatory elements Finder (HSF), to analyze this mutation motif. This powerful silencer can effectively suppress the interaction between U1 SnRNP and the receptor site, even in the case when the RNA shows a high splicing score.

Moreover, the impact of the intron 3’SS sequence on the splicing efficiency was analyzed. MaxEnt predicted that the +3A>G (+3G) mutation increased the 3’SS score from 13.09 (wild type) to 13.32. This mutation also weakened the inhibitory effect of potential ESS elements on splicing. Theoretically, these two factors should enhance gene expression. However, the experimental results show a significant decrease in Fluc expression (Figure 2C). The Mfold secondary structure analysis for pre-mRNA showed that the newly formed stem–loop in the mutant configuration, at a minimum free energy, obstructed the “AG site” of the 3’ SS, resulting in a diminished Fluc gene expression (Supplementary Figure S2).

According to the aforementioned result, it is clear that the efficient splicing of this artificial intron retention relies on a combination of various factors. To improve the Dox regulatory (Supplementary Figure S1: toxicity assay for optimal concentration) performance, we suggest choosing mutations with minimal initial expression levels. Figure 2C shows that the addition of Dox significantly enhances the expression level of mutant −1A+3G by 5.6-fold. However, its peak expression remained inadequate for subsequent detection. For subsequent application experiments, we chose the +3G mutation (with 4.5-times regulation) instead of the 1A+3G and named it CITetR.

**Figure 3:**
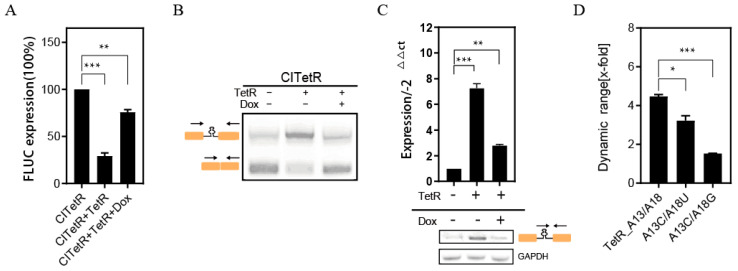
(**A**) Regulation of CITetR luciferase expression by TetR/Dox. (**B**) RT-PCR analysis using primer pairs targeting both exons. The lower band in the electrophoretic diagram suggests no intron retention and confirms proper Fluc expression. “+” indicates co-transfections with CITetR and LS-TetR or the addition of Dox; “−” indicates CITetR alone or no Dox. Arrows show the directions of the primers. Primer information is provided in Supplementary Figure S9. (**C**) qPCR and RT-PCR using primers targeting introns and exons, respectively. qPCR values were normalized to the CITetR group, with GAPDH as an internal control. (**D**) Effect of various mutations in aptamer sequences on Fluc dynamic regulation. Error bars indicate mean ± s. e. m (* *p* < 0.05, ** *p* < 0.01, and *** *p* < 0.001; unpaired Student’s *t*-test).

We co-transfected HeLa cells with CITetR and LS-TetR plasmids that encode the TetR protein. Following a 5 h incubation, we introduced 50 μM of Dox and assessed the luciferase activity 24 h after to confirm the splicing recovery process. Figure 3A illustrates that the exogenous TetR protein diminishes the luciferase activity to 28.0%, whereas the introduction of Dox reinstates the luciferase activity to 72.4% of the baseline level. This verifies that the CITetR switch proficiently suppresses spliceosome activity via an interaction with the TetR ligand and that Dox facilitates the dissociation of the ligand from the switch, albeit not entirely detaching TetR from the aptamer region.

Intron or exon primers were further used to assess intron retention visually, with the RT-PCR results in Figure 3B being consistent with the previous findings. The qPCR results show a significant reduction in intron-retention products after Dox treatment, although they are still 2.4-times higher than those of the negative control (Figure 3C). This suggests that Dox regulation did not achieve complete splicing. We hypothesize that this limitation may be attributed to TetR aptamer secondary structures, which affect the interaction between the spliceosomes on RNA, resulting in some inhibition of intron splicing [48,49]. 

Point mutations were also introduced to the key sites in the TetR aptamer sequence (Supplementary Figure S3). The dynamic regulatory capacity of the mutant sequence (A13>C/A18>G) was reduced by approximately 80% compared to the wild-type sequence (A13/A18) (Figure 3D). Thus, it can be concluded that the conservation of the aptamer sequence is essential for the effective recognition and participation of the ligand in regulating intron splicing [50]. 

#### 2.1.2. Constructing the CTM Module—Modularization and Feasibility Analysis of CITetR

**Figure 4:**
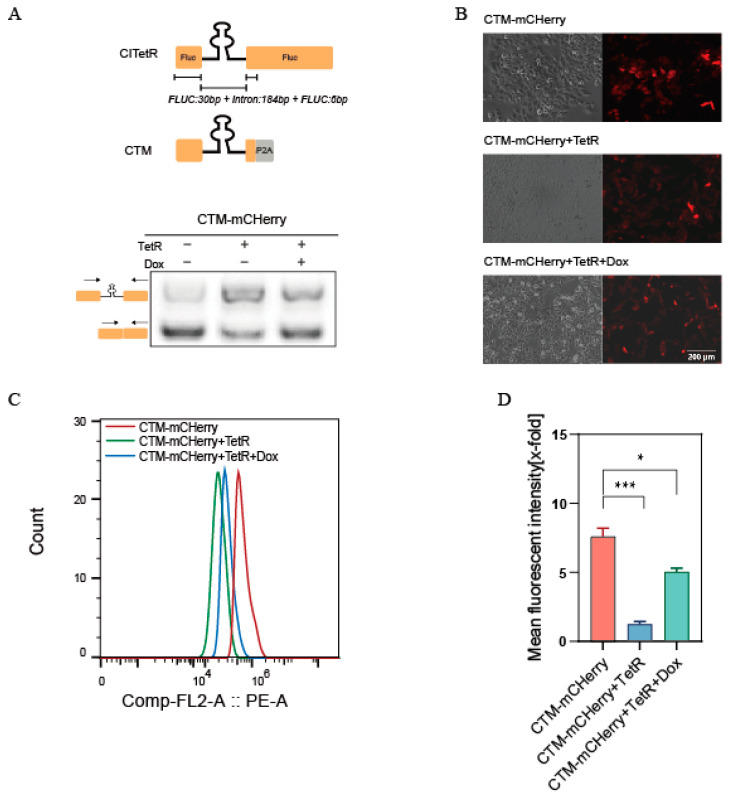
(**A**) RT-PCR analysis of CTM-mCHerry using primer pairs targeting both exons. The CTM’s construction: artificial chimeric intron (black) with flank sequence (orange) from Fluc (Fluc:30bp+Intron:184bp+Fluc:6bp). “+”:co-transfected with CTM-mCherry and LS-TetR or the addition of Dox; “−” indicates CTM-mCherry alone or no Dox. Arrows show the directions of the primers. Primer information is provided in Supplementary Figure S9. (**B**) Fluorescence expression of mCHerry was observed by fluorescence microscopy. Scale bar = 200 μm. CTM-mCHerry: CTM-mCHerry’s structure was transfected into Hela cell lines; CTM-mCHerry+TetR: CTM-mCHerry’s structure was transfected into Hela cell lines that can stably express the TetR protein. (**C**,**D**) Peak plots and histograms, respectively, displaying the average fluorescence intensities of three CTM-mCHerry experimental groups. Values between groups are normalized to the CTM-mCHerry+TetR group. The error bars indicate the mean ± s. e. m. (* *p* < 0.05 and *** *p* < 0.001; unpaired Student’s *t*-test).

Considering that switch is susceptible to various factors in different cellular environments, we modularized the entire CITetR system and transformed it into a versatile device that can regulate any gene of interest. Previous screening results have shown that CITetR flanker sequences are crucial for intron splicing. We thus retained the upstream 30 bp and downstream 6 bp sequences flanking the Fluc intron in the CITetR structure as a complete module, called the CTM. This module was inserted below the mCHerry gene start codon ATG. The 19 amino acid self-cleaving peptide P2A sequence upstream of the mCherry reading frame induces ribosomal skipping during protein translation, minimizing gene expression effects. The regulatory process governing CTM-mCHerry is demonstrated in Supplementary Figure S4. Flow cytometry revealed that the mean fluorescence intensity of co-transfected CTM-mCherry and LS-TetR reduced by 6.4 times due to the TetR protein interaction, while Dox increased it by 4.2 times. RT-PCR confirmed Dox’s effect on expression (Figure 4). The CTM architecture, therefore, exhibits consistent regulatory efficiency across diverse target gene environments.

#### 2.1.3. Long-Term and Reversible Dynamic Regulation of CTM-mCHerry

A TetR-Hela cell line stably expressing CTM-mCHerry was established to evaluate whether Dox can maintain long-term, reversible dynamic regulation in the CTM system. The variations in mCherry transcription/translation levels were monitored over 8 days by alternately administering or withdrawing 50 μM of Dox every 48 h.

**Figure 5:**
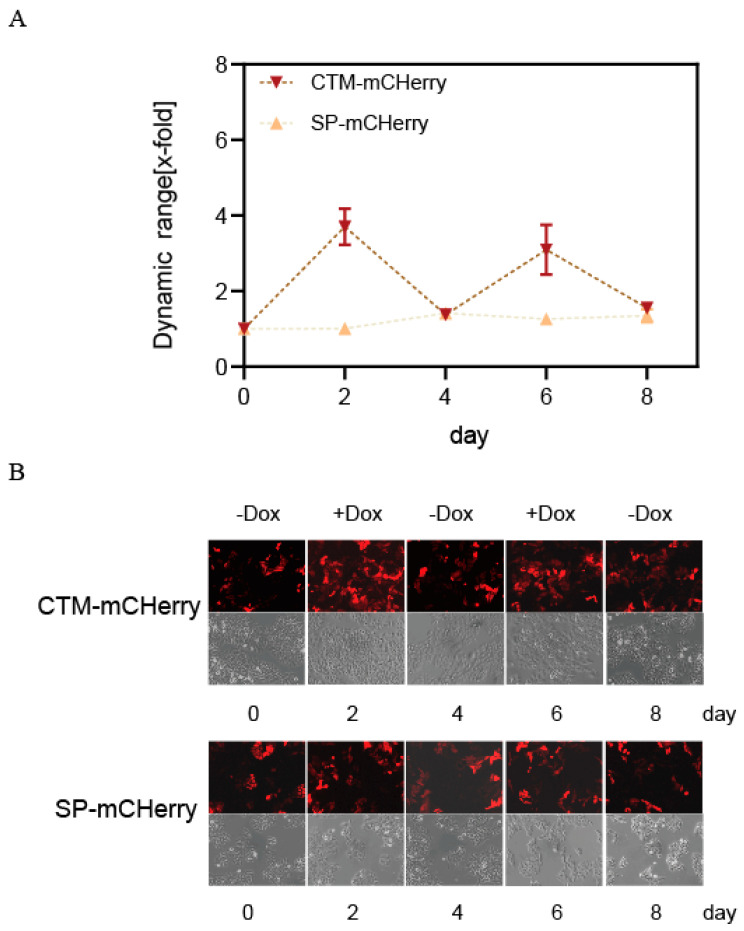
(**A**,**B**) The long-term and reversible dynamic regulation of CTM-mCHerry was assessed over an 8-day period by monitoring changes in the Dox concentration, with validation through RT-PCR or analysis of fluorescence intensity variations. Primer information is provided in Supplementary Figure S10. SP-mCHerry is a negative control without a switch. The error bars indicate the mean ± s. e. m.

Figure 5 illustrates that the experimental group exhibited optimal RNA-level regulation 48 h after Dox addition, while this regulatory capacity diminished 48 h after Dox removal. The reintroduction of Dox fully restored the regulatory function. This suggests that Dox concentration changes confer a continuous and dynamic regulatory capacity on the system. Thus, the CTM-mCHerry structure presented a reversible and persistent pattern of gene expression, as indicated by the fluctuations in mCherry fluorescence. This pattern is characterized as “ON–OFF–ON”, thereby opening up a wide range of potential applications for TetR in controlling intron splicing.

#### 2.1.4. The CTM Module Regulates the Functionality of the HSV-TK/GCV System

**Figure 6:**
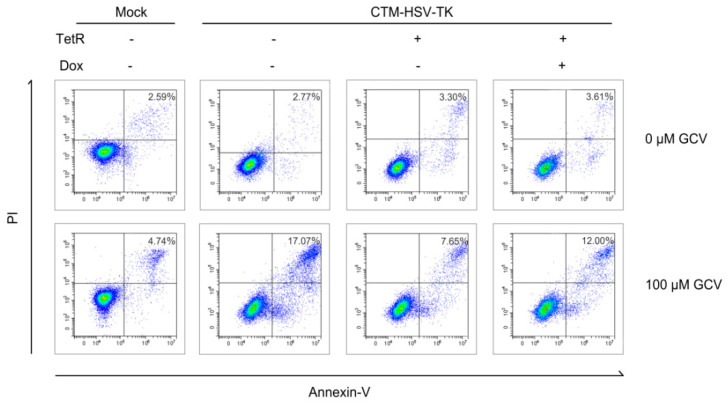
CTM-HSV-TK controls the HSV-TK/GCV system. CTM-HSV-TK and TetR protein particles were co-transfected into Hela cells and treated with 50 μM of Dox for 4–6 h. The effect of Dox on flow cytometry was observed after 36 h. The addition of the TetR ligand significantly attenuated the rate of apoptosis, which was subsequently restored upon Dox induction. The PE Annexin V/7-AAD apoptosis detection kit was used to stain the cells and control cell colors determine threshold localization. Living cells were negative for Annexin V and 7-AAD, apoptotic cells were positive for Annexin V but negative for 7-AAD, and dead cells were positive for both.

We further investigated the potential of the CTM switch in modulating the HSV-TK/GCV system with Dox, which is crucial to effectively control and ensure the safety of TK suicide gene expression (Supplementary Figure S5). The CTM module was integrated into the HSV-TK gene and transiently transferred to TetR-Hela cells. Supplementary Figure S6 confirms the efficacy of Dox in regulating intron retention. Figure 6 demonstrates that TetR significantly decreased the cell apoptosis rate from 17.07% to 7.65%, subsequently rising to 12% following Dox treatment (effective GCV concentration of 100 μM), indicating that this modular device is versatile with respect to various ligand inputs and regulatory targets, thus enhancing its potential for therapeutic applications.

### 2.2. Dox-Dependent Switch-Off Device 

#### 2.2.1. Principle and Design of Dox-Dependent Switch-Off Device 

During our research, we unexpectedly discovered that shifting the TetR aptamer in the CTM module 2 base pairs to the right within the intron sequence resulted in regulatory behavior that was entirely contrary to that of the CTM: after incorporating this modified sequence into the eGFP exon, in the absence of Dox, the gene expression increased, while the addition of Dox reduced eEGFP expression. This new structure has been designated as “C2ITetR”.

**Figure 7:**
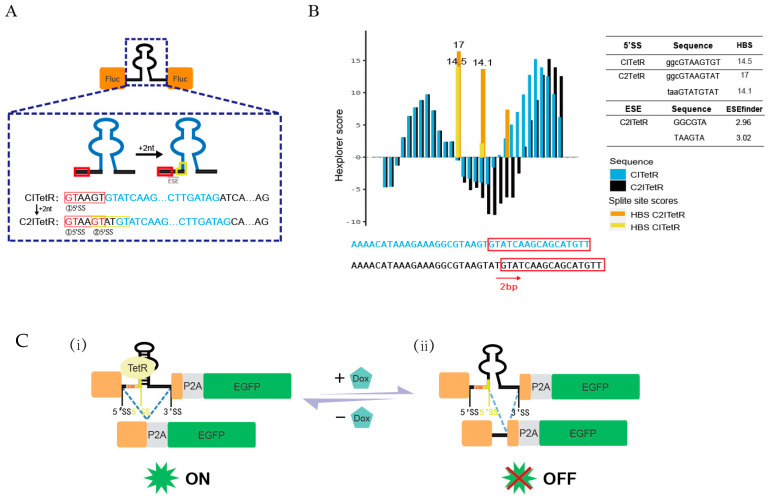
Schematic of the splice-site alterations of C2ITetR. (**A**) Schematic representation of the generation of a secondary 5′ splicing site in the chimeric intron. Red box: upstream 5’SS splicing module; yellow box: downstream 5’SS splicing module; blue: TetR adapter area. (**B**) HEXplorer and HBS analysis for CITetR/C2ITetR. (**C**) (**i**) The absence of Dox leads to the spatial blocking of the downstream 5’SS by the TetR ligand, resulting in upstream 5’SS splicing selection and intron retention, thereby maintaining normal gene expression. (**ii**) The addition of Dox alters the pre-mRNA splicing site, favoring the choice of the second 5’SS without TetR blocking. Consequently, the reading frame of the target gene is disrupted, and gene expression is inhibited. Short black sequence: upstream 5’SS splicing site; bright-yellow sequence: downstream 5’SS splicing site.

The HBond score (HBS) [37], an online tool for predicting the 5’SS based on the hydrogen bond model of the interaction of U1 snRNA and its binding site, was utilized to identify subtle base changes in C2ITetR. The predictions revealed a second valid 5’SS was located 4 bp downstream of the original site, with a splicing score of 14.1 compared to 17.0 for the upstream site (Figure 7A,B). It is worth noting that splicing regulatory elements (SREs) play a crucial role in splicing sequence recognition [51]. Therefore, we used the ESE finder for splicing enhancer sequence analysis and found an ESE enhancer site with SR protein-binding capabilities (splicing enhancer element) located between two 5’SSs. As previously reported [51,52], these sites exhibit a significant directional bias: it inhibits upstream splicing while enhancing downstream splice-site usage. 

Therefore, we speculate that Dox addition prevents the TetR protein from binding to the aptamer structure, resulting in pre-mRNA having more freedom to select downstream splicing sites and leading to a shift in the reading frame. Conversely, in the absence of Dox, TetR protein-induced aptamer structural changes limit the spatial selection of the downstream 5’SS, preferring the upstream site, which guarantees the proper expression of genes (Figure 7C). 

**Figure 8:**
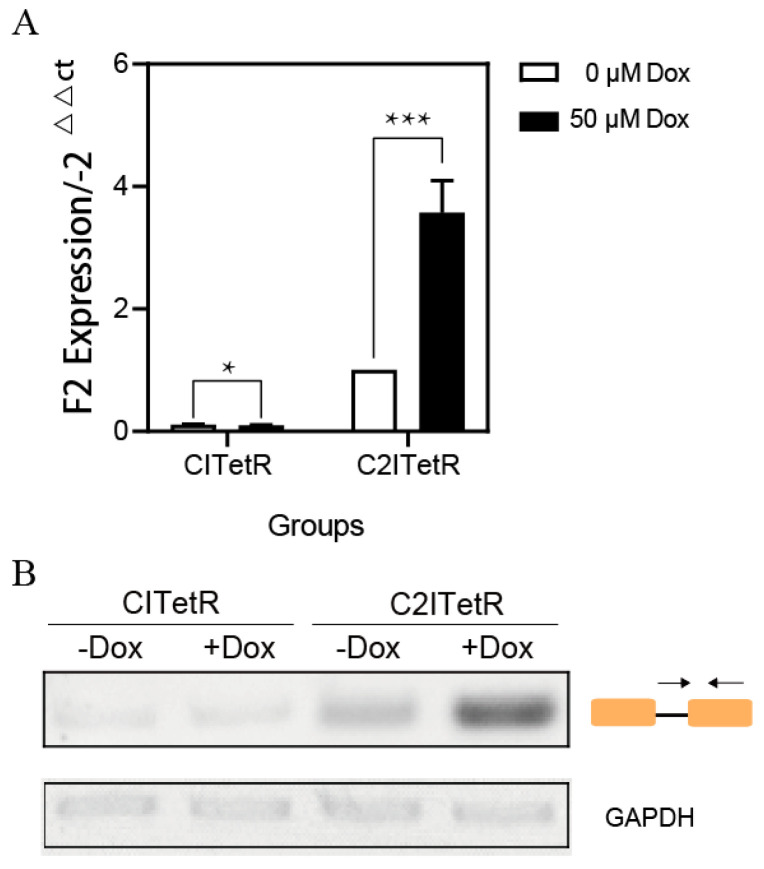
(**A**) Intron-retention sequence of C2ITetR was analyzed using qPCR in the presence of Dox or without Dox. The upstream primer from the intron sequence and the downstream primer from the P2A sequence. CITetR was used as a negative control. (**B**) The intron-retention sequence of CITetR/C2ITetR was analyzed using RT-PCR, with GAPDH serving as an internal reference. Primer information is provided in Supplementary Figure S9. (* *p* < 0.05 and *** *p* < 0.001; unpaired Student’s *t*-test).

We further quantitatively identified the splicing products of C2ITetR mRNA using sequence primers from the intron. The data in Figure 8 show that Dox induction increases C2ITetR spliced isoforms by 3.6-fold, supporting our hypothesis: The addition of Dox improves the likelihood of selecting a secondary splice site. 

#### 2.2.2. Structure Optimization of C2ITetR 

Based on the aforementioned observation, it is feasible to construct a Dox-based “off-type” reversible switch using C2ITetR for regulating gene expression. However, its regulatory efficiency requires improvement. To optimize this, we focused on two key aspects: enhancing basal fluorescence expression in the absence of Dox and reducing expression leakage after Dox addition.

**Figure 9:**
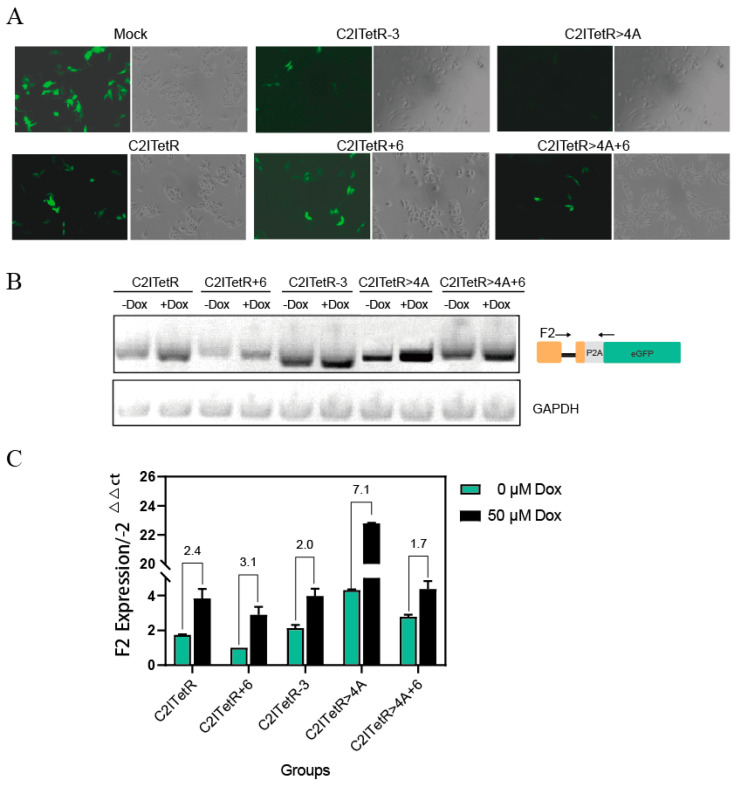
(**A**) The impact of various stem lengths or mutations in the aptamer on eGFP gene expression without Dox. The fluorescence leakage conditions vary among different structures in the C2ITetR “on” state under the same cell density, with C2ITetR>4A exhibiting the least fluorescence leakage. (**B**) RT-PCR analysis of the intron-retention sequence in various mutant modules with or without Dox. GAPDH serves as the internal reference. Arrows show the direction of the primers. Primer information is provided in Supplementary Figure S9. (**C**) qPCR analysis of the intron-retention sequences in various mutant modules with or without Dox, normalized by the C2ITetR group. The error bars indicate the mean ± s. e. m.

Firstly, according to the previous study [48,49], splicing accessibility is inversely related to the stability of the RNA secondary structure. Therefore, we attempted to enhance pre-mRNA stability by increasing the aptamer stem length, thereby reducing the selection of the downstream 5’SS and promoting the selection of the upstream 5’SS. This approach reduces intron retention and improves fluorescence expression. We modified the aptamer stem length by reducing it by 3 bp (named C2ITetR-3) or increasing it by 6 bp (named C2ITetR+6). Supplementary Figure S7 and Table 1 show the thermodynamic free energy (ΔG) of these structures in their free state. These three constructs were transfected into HeLa cells stably expressing the TetR protein. After 24 h, the fluorescence intensity trend was observed as C2ITetR+6 > C2ITetR > C2ITetR-3 (Figure 9A), consistent with the predictions. RT-PCR analysis revealed that the intron-retention rate for C2ITetR+6 was significantly lower than for the other two constructs (Figure 9B), which corresponded to the eGFP intensity changes observed under fluorescence microscopy. This indicates that increasing the aptamer stem length enhances access to the first 5’ splicing site.

**Figure 10:**
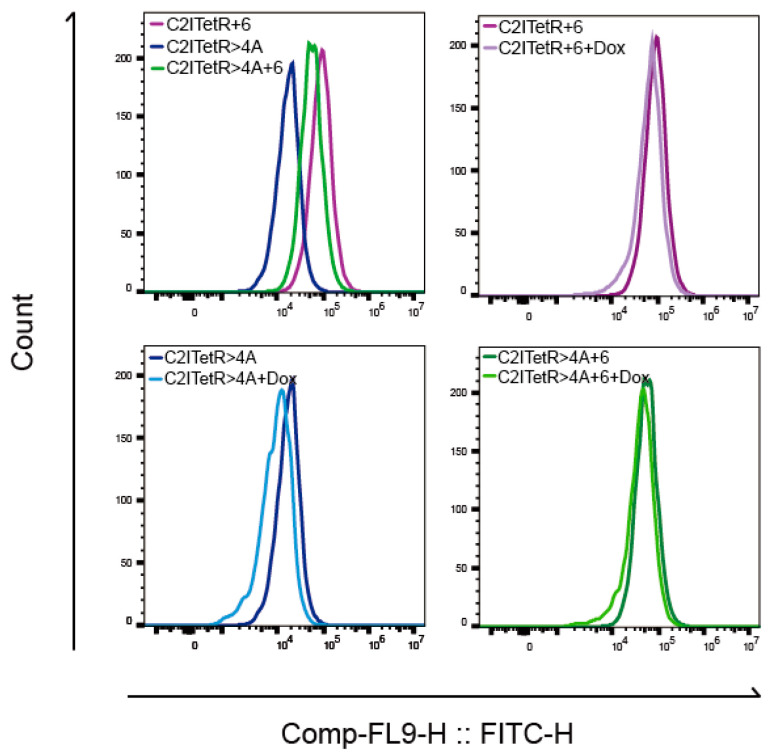
Flow cytometry analysis was performed on various mutant modules. The average fluorescence intensity histogram of each mutant element in the absence of Dox is presented in the upper-left panel. The lower-left, upper-right, and lower-right panels represent the comparative average fluorescence intensities of C2ITetR>4A, C2ITetR+6, and C2ITetR>4A+6, respectively, with or without Dox. The regulatory effect of Dox on C2ITetR>4A is superior.

Despite these modifications, Dox’s maximum regulatory effect was only 3.1-fold (Figure 9A and RT-PCR results). To improve regulation further, we introduced mutations at positions +1 to +6 downstream of the second 5’ splicing site. Using HBS screening, we found that the mutant C2ITetR>4A had the highest splicing score of 17.4 (Supplementary Figure S8). Dox treatment raised the regulatory ratio of C2ITetR>4A by 7.1 times, according to RT-PCR analysis (Figure 9B,C). The regulatory effects of Dox on the initial fluorescence intensity and switching function of eGFP were also validated by flow cytometry (Figure 10). Thus, we chose C2ITetR>4A as the “off switch “in the experiments that followed.

### 2.3. Establishment of an RNA Double-Switch Cascade Circuit Based on CTM/C2ITetR>4A

Previous data on the regulation of mCherry by the CTM module indicate that the maximum fold-change in expression upon the addition of Dox was merely 4.5-fold, presenting a challenge for the optimization of regulatory efficiency. To achieve this, we intend to further diminish the basal expression of the system in the “OFF state” by concurrently targeting translation and transcription levels. Consequently, we engineered an RNA circuit utilizing a cascade effect by combining the CTM with C2ITetR>4A.

**Figure 11:**
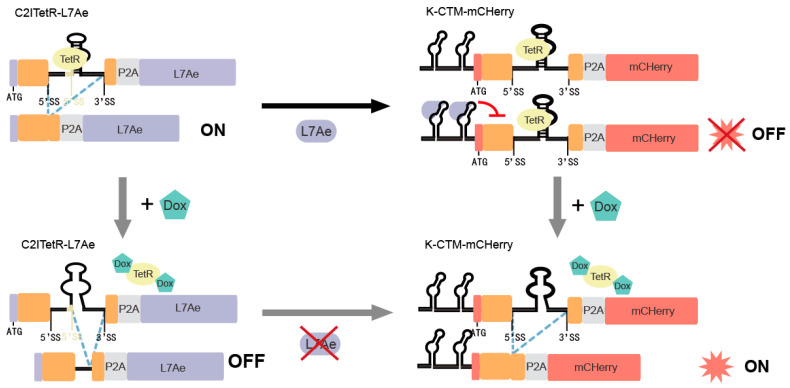
Schematic diagram of the cascade regulation of mCHerry through the combination of K-CTM-mCHerry and C2ITetR-L7Ae.

As illustrated in Figure 11, the L7Ae gene is placed downstream of the “off-type” switch CI2TetR>4A (CI2TetR-L7Ae), while the K-turn sequence is located in the 5’UTR region of the “on-type” switch (K-CTM-mCherry). The lack of external Dox kept the CI2TetR-L7Ae switch in the “off state”, increasing the L7Ae protein. This allows L7Ae to bind to the K-turn region in the 5’ UTR of K-CTM-mCHerry. This configuration is expected to result in a more stringent mCHerry regulation under the influences of both the L7Ae protein and Dox. In contrast, the introduction of Dox decreases the expression of L7Ae and facilitates the dissociation of TetR from RNA, allowing splicing to be restored in K-CTM-mCHerry, and consequently restoring mCherry expression.

**Figure 12:**
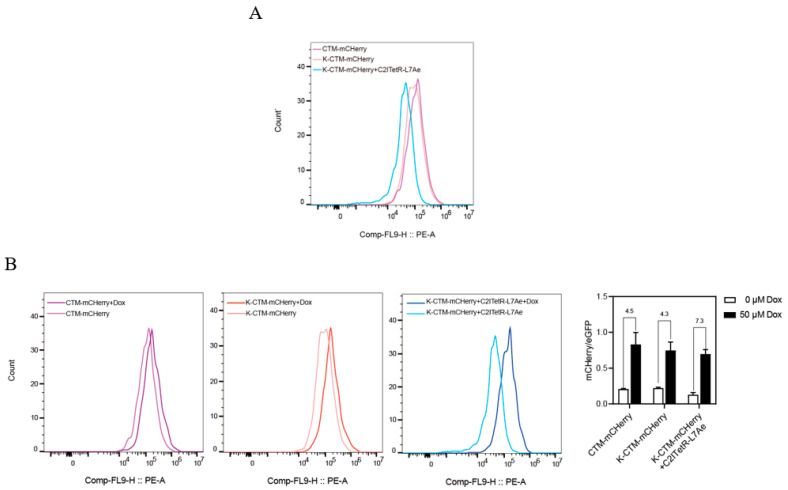
(**A**) The initial expression leakage inhibition effects of the K-turn structure alone and cascaded combined switches on mCHerry were compared using flow histograms in the absence of Dox. (**B**) The regulatory effect of the K-CTM-mCHerry and C2ITetR-L7Ae cascade switches on mCHerry at a concentration of 50 μM Dox, with eGFP serving as the internal control parameter. The error bars indicate the mean ± s. e. m.

The two regulatory modulator systems were introduced into HeLa cells stably expressing TetR, with cells transfected with CTM-Cherry and K-CTM-mCherry alone serving as the controls. As shown in Figure 12A, fluorescence analysis revealed that, in the absence of Dox, the K-turn structure exerted a moderate inhibitory effect on the initial expression of mCherry. Compared to the independent-switch CTM, the combination switch (C2ITetR-L7Ae + K-CTM-mCherry) significantly reduced the initial expression of mCherry.

Upon the addition of Dox, the cascade effect of the combined switch markedly enhanced the regulatory efficiency of mCherry expression (Figure 12B,C), achieving up to a 7.3-fold increase. This demonstrates that the dual regulation by the L7Ae protein and Dox can effectively address the leakage issue of CTM-mCherry in the initial state, thereby improving the rigor of regulation. 

## 3. Discussion and Conclusions

In this study, an artificially constructed RNA element was employed to regulate the alternative splicing of Pre-mRNA in human cells for the purpose of controlling gene expression. We constructed a TetR ligand-regulated “on-switch” CTM and an “off-switch” C2ITetR>4A. Based on the precise recognition of exon–intron boundaries at 5’SS and 3’SS single nucleotides [50,52], a series of mutations was engineered to enhance splicing efficiency, while the splicing regulatory elements (SREs) showed their more powerful influence on the spliceosome [49]. For example, the -4G>A mutation in CITetR, although possessing a high splicing score, markedly diminishes the splicing efficiency due to the introduction of an exonic splicing silencer (ESS). The primary contribution of SREs is the identification of an ESE between the two 5’ splice sites in C2ITetR. This discovery can modify the selection of splicing sites at the 5’ splice site, thereby becoming a key mechanism for reversing the switch.

Additionally, the thermodynamic stability of pre-mRNA secondary structures and its impact on adjacent splicing-site recognition must not be ignored. This is exemplified by modifications to the C2ITetR aptamer stem length and the CITetR+3A>G mutation. Surprisingly, displacing the aptamer in the module gene by merely two base pairs reversed the switching effect, suggesting that splicing factors interact in an intricate rather than merely additive manner. Consequently, identifying optimal equilibrium is essential for improving splicing efficacy. In addition, during the optimization of the C2ITetR module, increasing the aptamer stem length (C2ITetR+6) and enhancing the splicing score (C2ITetR>4A) can each independently improve the efficiency of splicing regulation. However, when both changes are implemented simultaneously (C2ITetR>4A+6), regulatory efficiency is significantly reduced, achieving only a 1.7-fold improvement (Figure 9C). This indicates that achieving the appropriate balance between gene expression levels and riboswitch regulatory capacity is crucial for optimizing the switch performance.

Based on the “plug-and-play” principle of components, such as switches in synthetic biology, we not only validated the effectiveness of CTM switches in reporter genes, but also applied them to functional genes. By inserting the switch into the exon of the HSV-TK gene, gene expression can be conditionally regulated by external molecule concentrations, enabling the controlled induction of tumor cell apoptosis using small-molecule drugs.

We further integrated two types of switches to construct a double-switched RNA cascade system based on intron splicing. This system better controls target gene leakage in the absence of Dox and simultaneously activates and inhibits the splicing system in the presence of Dox. A single ligand can engage with both systems, helping to reduce crosstalk between different aptamer switching elements and minimize unpredictable effects on gene expression, thereby achieving higher-dynamic-range gene expression regulation. This intron-splicing-based RNA circuit effectively controls gene expression and, when combined with various aptamers, allows small-molecule drugs, like Dox or inducible drugs, to reversibly activate translation. Thus, it provides a promising tool for mammalian synthetic biology, facilitating the design of more user-friendly, cell-type-specific, and conditional RNA circuits.

During the experiment, we acknowledged that various factors affect the precise splicing of Pre-mRNA into mature transcripts. These factors should be considered collectively rather than in isolation. Given the established complexity of alternative splicing processes [53], different splicing prediction software were used to optimize splicing sites and regulatory functions throughout the experiment. Numerous splice-site prediction programs currently in use typically begin with a single factor. The splicing ability of RNA is predicted, for instance, by the strength of ESEs/ESSs or ISEs (intron splicing enhancers)/ISSs (intron splicing silencers) and the splicing score of the cis-acting element 5’SS/3’SS; yet, the impact of a secondary structure on splicing is sometimes neglected, and the modification of RNA switching elements is not taken into account. Therefore, in the forthcoming experiments, we aim to collect more comprehensive splicing regulation data, integrate the advantages of different prediction system algorithms, and establish a more accurate computational model to predict alternative splicing. Deep learning has recently been used in some studies to explain how primary sequences contribute to splice-site selection [54]. The model will establish a theoretical foundation for an in-depth examination of the mechanisms and regulation of alternative splicing in diseases, with wider implications for the treatment of associated conditions.

## 4. Materials and Methods

### 4.1. Cell Culture

The Hela cells (Cellcook, Guangzhou, China) were cultured in a 5% CO_2_ incubator at 37 °C with 10% (*v/v*) fetal bovine serum (ExCell Bio, Suzhou, China) and 1% (*v/v*) penicillin–streptomycin. Cell passage was performed every 2–3 days, ensuring the use of cells within a maximum of 40 generations.

### 4.2. Plasmid Construction

The plasmid assembly enzymes (Ex Taq DNA polymerase/T4 ligase) and restriction enzymes were obtained from TaKaRa Biotech (Shiga, Japan). Clone-in enzymes were acquired from Vazyme Biotech (Nanjing, China) and employed according to the manufacturer’s instructions.

Oligonucleotides were synthesized by Sangon Biotech (Shanghai, China). Primer sequences and PCR conditions are available upon request. EcoRI/HindIII restriction sites were employed for cloning Fluc into pcDNA3.1 (+), resulting in a pcDNA3.1+/Fluc construct. The expression of Fluc was driven by a CMV promoter followed by a bgl polyA sequence. Homologous recombination was then employed to insert the complete intron gene into Fluc, generating the pcDNA3.1+/Fluc/CI construct. Subsequently, a TetR aptamer was inserted downstream of the 5’SS of the intron region in order to create the pcDNA3.1+/Fluc/CITetR construct. Shifting the position of the TetR aptamer’s insertion into the intron region by 2nt toward the right resulted in pcDNA3.1+/Fluc/C2ITetR.PcDNA3.1+/Fluc/CTM-mCHerry/HSV-TK and PcDNA3.1+/Fluc/C2ITetR-L7Ae constructs being generated through the simultaneous homologous recombination of amplified P2A and mCHerry/HSV-TK/L7Ae fragments with a CITetR/C2ITetR module (the intron containing the TetR aptamer and 36 nucleotides from Fluc) in pcDNA3.1+/Fluc/CITetR and pcDNA 3.1+/Fluc/C2ITetR, respectively.

By incorporating the EcoRI and XhoI restriction sites downstream of the cmv promoter, a plasmid encoding the ligand protein was successfully constructed. Simultaneously, the N-terminal of the TetR protein was modified with a nuclear localization sequence derived from SV40, resulting in the generation of NRS-TETR possessing nuclear localization signal (NLS) functionality.

### 4.3. Dual-Luciferase Reporter Assay

Each well plate received 60,000 HeLa and 30,000 293T cells. Following the manufacturer’s instructions, Hieff Trans^®^ liposomal transfection reagent (Yeasen Biotech, Beijing, China) was used to co-transfect 100 ng reporter cell DNA plasmid and 300 ng TetR plasmid after 24 h. The media were replaced with a new medium containing or without 50 μM of doxycycline (Sigma-Aldrich, St. Louis, MO, USA). After 4–6 h, detection was carried out using the dual-luciferase reporter gene method provided by the Beijing Esen Biotechnology Company in China. The TECAN (Männedorf, Switzerland) Spark multi-mode microplate reader measured each well’s light signal. Firefly–Renila luminescence ratios assessed FLuc/RLuc relative luciferase activity. Additionally, the mean and standard deviation were calculated for three replicates simultaneously while utilizing the vector without an aptamer as a reference for normalization purposes. Each experiment was repeated three times.

### 4.4. RT-PCR/qPCR Analysis

After seeding 120,000 HeLa cells per well in a 12-well plate, 100 ng reporter DNA and 300 ng TetR plasmids were co-transfected after 24 h. All transfections were performed using the Hieff Trans^®^ Liposomal Transfection Reagent (Yeasen Biotechnology, Beijing, China) according to the manufacturer’s instructions. After incubation for 4–6 h, the culture fluid was replaced with new media containing or without 50 μM of doxycycline (Sigma-Aldrich).

Next, the cells were grown at 37 °C in 5% CO_2_ for another 24 h before RNA extraction with TRIzol^®^ (Sangon, Shanghai, China). An electrophoresed gel of 500 ng of RNA in 1% (*w/v*) agarose tested RNA integrity. Intact RNA samples were subsequently stored at −80 °C in a refrigerator. Complete ex-gDNA synthesis was achieved using a reverse transcription kit (Accurate Biology, Changsha, China) and 1 μg of RNA with subsequent reverse transcription. Taq polymerase (TAKARA, Japan) was used to amplify cDNA under the following conditions: initial denaturation at 95 °C for 3 min, 30 s at 95 °C, 30 s at 60 °C, and 30 s at 72 °C, repeated 23 times. After amplifying, 3% agarose gel was used to examine the results.

The cDNA samples obtained by reverse transcription were subjected to qPCR analysis using the SYBR Green Pro Taq HS qPCR kit (Accurate Biology, Changsha, China) on a StepOnePlus instrument (Applied Biosystems, Waltham, MA, USA). The data were analyzed using the 2^−ΔΔCT^method based on at least three replicates from three independent experiments.

### 4.5. Fluorescence Microscopy

The 12-well plates were each inoculated with 120,000 Hela cells for microscopic observation, and glass coverslips were used to cover the wells. CITetR: 200 ng CTM-mCHerry reporter DNA plasmid: CTM-mCHerry+TetR/CTM-mCHerry+TetR+Dox: 200 ng CTM-mCHerry reporter DNA plasmid and 600 ng of TetR plasmid were transfected with 2 μL (Yeasen Biotechnology, Beijing, China) per well. The culture media were replaced after 4–6 h with fresh media with or without 50 μM of doxycycline (Sigma-Aldrich). The cells were cultured at 37 °C in an atmosphere containing 5% CO_2_ for 24 h. After two PBS washes and fixation (washing twice with PBS and fixing) with 4% paraformaldehyde (Biosharp, Beijing, China) for 15 min, the coverslips with cells were snapped onto slides. The slides were fixed onto a microscope (Zeiss, Germany) for cell imaging and image capture. The resulting pictures were quantified using ImageJ software Version 1.54m.

### 4.6. Flow Fluorescence Assay

eGFP and mCHerry fluorescence intensities were measured using CytoFlex S flow cytometry (Beckman Coulter). All measurements were performed in triplicate, and the mean and standard deviation were calculated based on normalization to eGFP. For eGFP and mCHerry, the excitation wavelengths were 488 and 561 nm, with emission filters at 520/35 and 610/20 nm, respectively. Flowjo v10.6.2 (TreeStar Inc., Ashland, OR, USA) was used to remove background fluorescence from untransfected cells in the cell population.

### 4.7. Apoptosis Flow Assay

The BBcell Probe Annexin V Apoptosis Detection Kit (BestBio Biotechnology, Nanjing, China) was used to detect apoptosis in transfected cells. After centrifugation, 24 h post-transfected cells were resuspended in 400 μL of 1X Annexin V binding buffer and stained with 5 μL of Annexin V-FITC and 5 μL of PI for 15 min on ice. To analyze 20,000 events, labeled cells were combined with 400 μL of binding buffer and examined with flow cytometry.

## Figures and Tables

**Table 1:** Thermodynamic table of TetR aptamers with different stem lengths (various numbers of complementary pairs).

Complemenary Base Pairs	Sequence	ΔG (kcal/mol)
−4	GTATCAGCATGTTATGGGTCATCACAGACCAGAGAAAAGATAC	−7.5
−3	GTATCCAGCATGTTATGGGTCATCACAGACCAGAGAAAAGGATAC	−10.4
−2	GTATCGCAGCATGTTATGGGTCATCACAGACCAGAGAAAAGCGATAG	−11.8
−1	GTATCAGCAGCATGTTATGGGTCATCACAGACCAGAGAAAAGCTGATAG	−13.3
0	GTATCAAGCAGCATGTTATGGGTCATCACAGACCAGAGAAAAGCTTGATAG	−15.3
+1	GTATC**A**AAGCAGCATGTTATGGGTCATCACAGACCAGAGAAAAGCTT**T**GATAC	−16.2
+2	GTATC**AG**AAGCAGCATGTTATGGGTCATCACAGACCAGAGAAAAGCTT**CT**GATAC	−19.3
+3	GTATC**AGT**AAGCAGCATGTTATGGGTCATCACAGACCAGAGAAAAGCTT**ACT**GATAC	−20.2
+4	GTATC**AGTC**AAGCAGCATGTTATGGGTCATCACAGACCAGAGAAAAGCTT**GACT**GATAC	−23.2
+5	GTATC**AGTCG**AAGCAGCATGTTATGGGTCATCACAGACCAGAGAAAAGCTT**CGACT**GATAC	−25.7
+6	GTATC**AGTCGC**AAGCAGCATGTTATGGGTCATCACAGACCAGAGAAAAGCTT**GCGACT**GATAC	−28.6
+7	GTATC**AGTCGCC**AAGCAGCATGTTATGGGTCATCACAGACCAGAGAAAAGCTTG**GCGACT**GATAC	−31.5

Note: Italic and underlined: the second 5 ‘SS related base; Bold: Add base pairs.

## Data Availability

All relevant data are within the paper and Supplementary Materials.

## Supplementary Materials

The following are available online at https://www.mdpi.com/article/10.3390/ijms26031163/s1.

## Abbreviations

AS: Alternative splicing; Dox: Doxycycline; 5′SS: 5’ splice site; 3′SS: 3’ splice site.
