# Supplementary material for: The Establishment of Artificial RNA Cascade Circuits for Gene Regulation Based on Doxycycline-Induced Pre-mRNA Alternative Splicing
Source: Int J Mol Sci. 2025 Jan 29;26(3):1163. doi: 10.3390/ijms26031163 (PMC11817826; doi:10.3390/ijms26031163)
Supplement: Supplementary file 1 [file ijms-26-01163-s001.zip › ijms-3403431-supplementary.pdf]

The Establishment of Artificial RNA Cascade Circuits for Gene Regulation Based on Doxycycline-induced Pre-mRNA Alternative Splicing

Supplementary Information

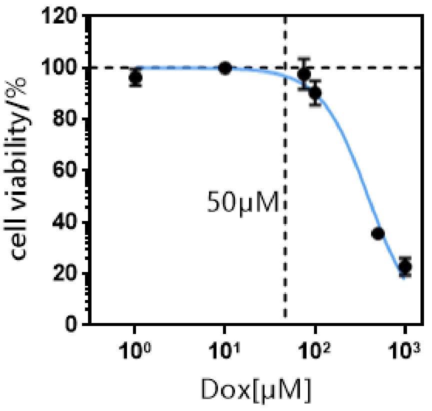

Figure S1 Dox toxicity assay for HeLa cells.

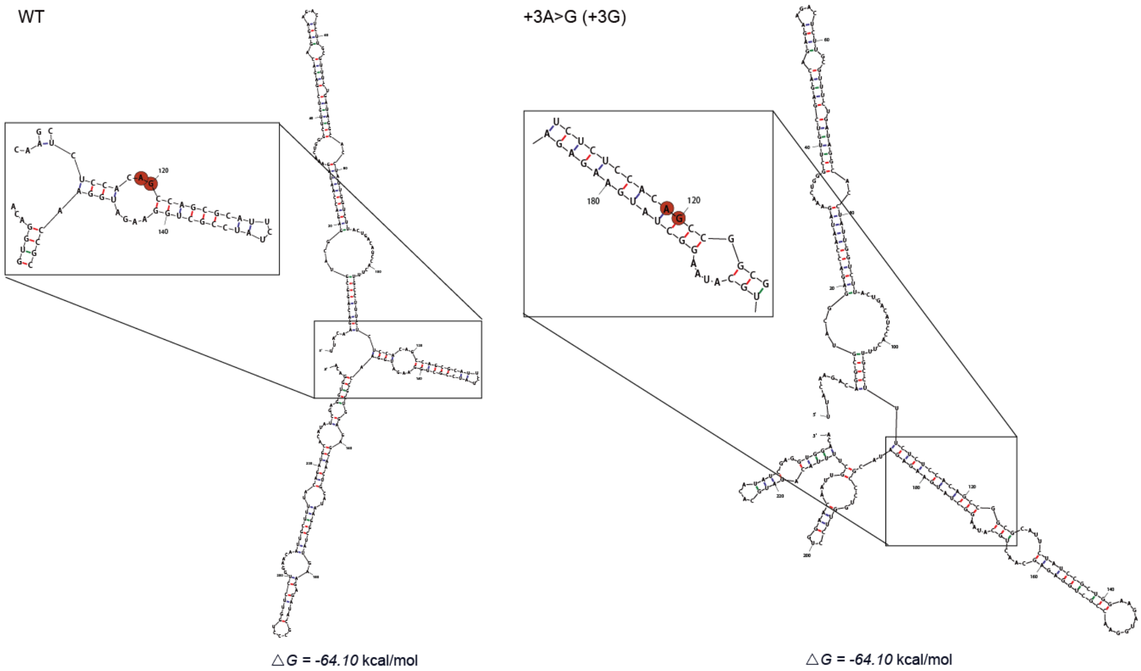

Figure S2 Secondary Structure Prediction of the 3' Splice Site in CITetRd (WT) and Mutant CITetRd+3A>G (+3G) Using Mfold. The results revealed that the newly formed stem-loop in the mutant structure, at minimum free energy, blocked the AG site (highlighted in red) of the 3' splice site , leading to decreased Fluc gene expression under initial conditions.

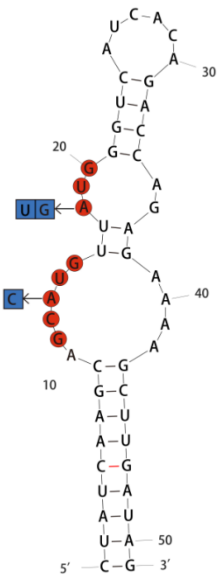

Figure S3 Schematic of TetR aptamer secondary structure. Key bases are shown in red, and mutations at positions 13A （A13>C）and 18A（A18>G) at this marker position are highlighted in blue.

A

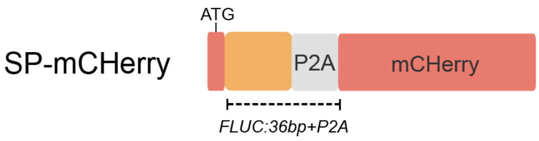

B

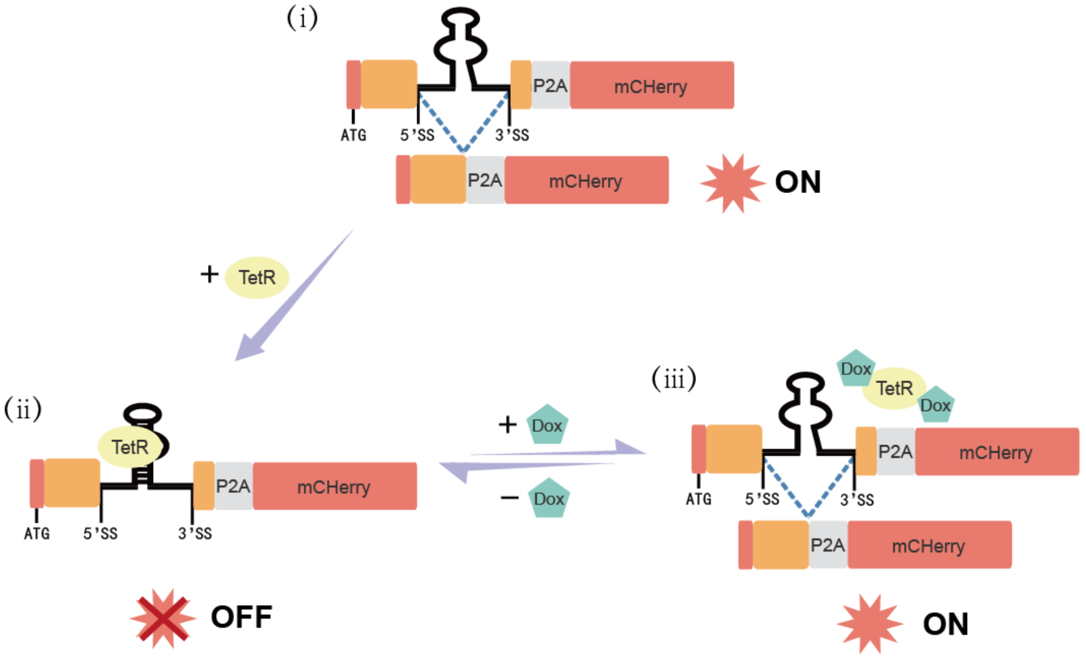

Figure S4 A.Schematic representation of SP-mCHerry. The retention of the upstream 30 bp and downstream 6 bp sequences of CITetR Fluc exons(orange) to preserve the splicing environment of introns， with the mCHerry gene ATG (red) inserted alongside the P2A sequence. The structure serves as a positive control for CTM-mCHerry, excluding CITetR switches, introns, and TetR aptamer sequences.

B. Schematic diagram of of Dox regulation for intron splicing in CTM-mCHerry. (i) in the absence of TetR, both intron splicing and mCHerry expression were normal; (ii)The addition of TetR inhibited splicing, leading to intron retention and decreased mCHerry expression; (iii) Dox induced the separation of TetR from its aptamer, restoring normal splicing and increasing mCHerry expression.

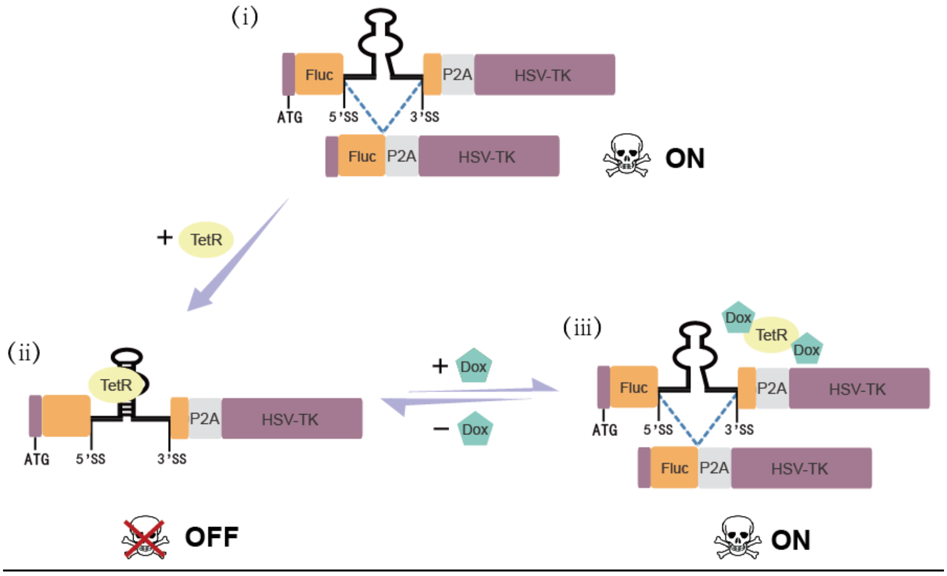

Figure S5 Schematic diagram of of Dox regulation for intron splicing in CTM-HSV-TK. The principle is similar to what is shown in the figure 3.

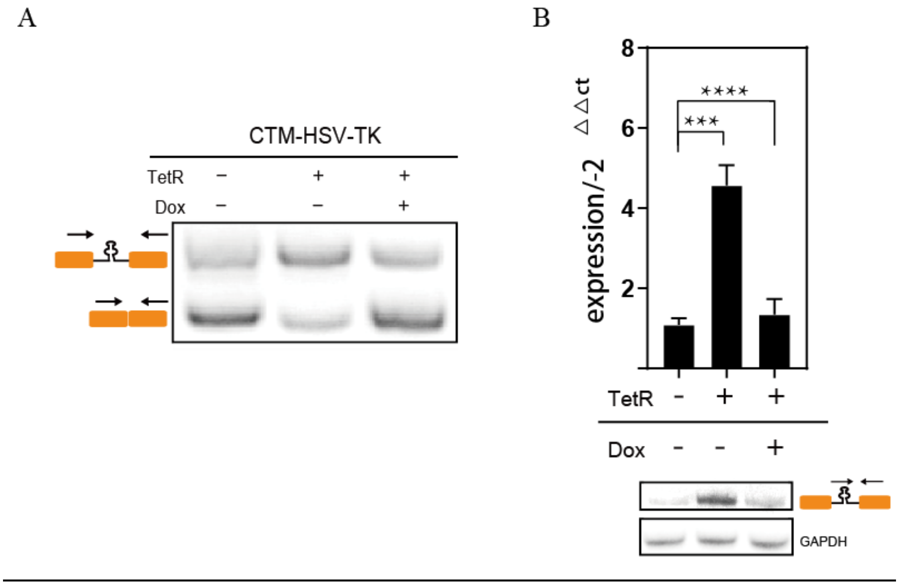

Figure S6 Intron or exon primers employed to visually assess intron retention in CTM-HSV-TK,The intron retention were significantly increased with TetR, while reduced after treatment Dox.”+" indicates co-transfected with CTM-HSV-TK and LS-TetR or addition of Dox ; "-" indicates CTM-HSV-TK alone or no Dox .Primers information is provided in Supplementary Figure.9.

A. RT-PCR analysis was performed using primer pairs targeting both exons,

B. qPCR and RT-PCR using primes targeting intron and exon respectively. The qPCR values were normalized to CTM-HSV-TK group, with GAPDH as an internal control.The error bars indicate the mean±s. e. m (***p < 0.001,****p<0.0001; unpaired Student’s t-test).

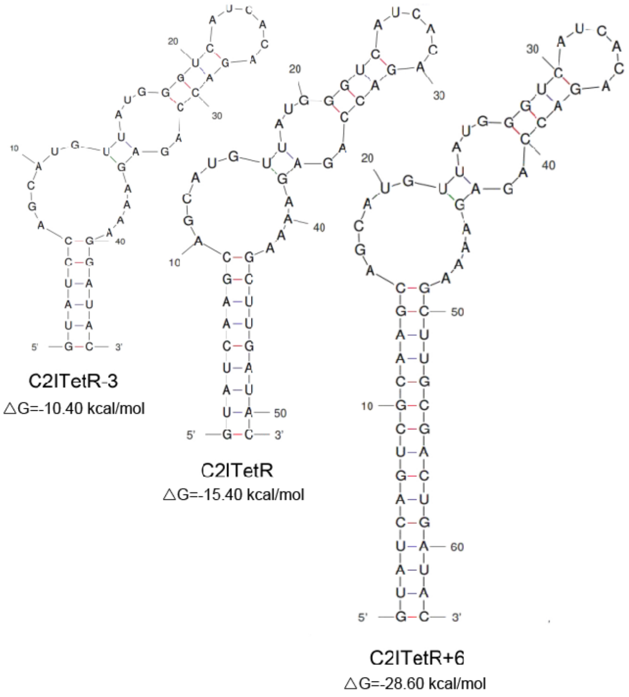

Figure S7 Schematic diagram of various stem lengths in the aptamer structures of C2ITetR-3, C2ITetR and C2ITetR+6.

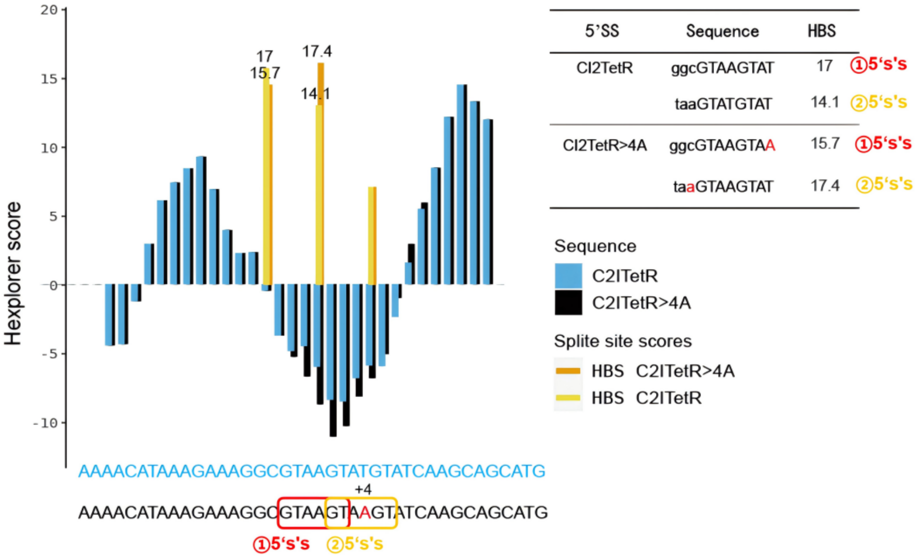

Figure S8 HBS analysis for C2ITetR and mutant C2ITetR>4A splicing. C2ITetR>4A: a substitution of A for T at position +4 bp downstream of the second 5'SS

Red box: upstream 5' SS splicing module; Yellow box: downstream 5' SS splicing module;

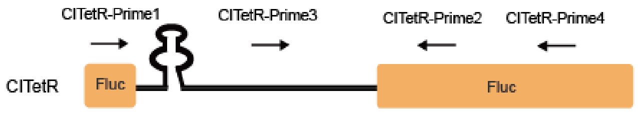

CITetR-Prime1	CCACCATGGAAGACGCCAAA
CITetR-Prime2	GCGTAAGTGATGTCCACCTCG
CITetR-Prime3	CTTGCGTTTCTGATAGGCACC
CITetR-Prime4	TCATAGCTTCTGCCAACCGA
GAPDH-1	GATTCCACCCATGGCAAATTC
GAPDH-2	CTGGAAGATGGTGATGGGATT

CITetR-Prime1 and CITetR-Prime2 were used for Figure 3B, while CITetR-Prime3, CITetR-Prime4, GAPDH-1, and GAPDH-2 were used for Figure 3C.

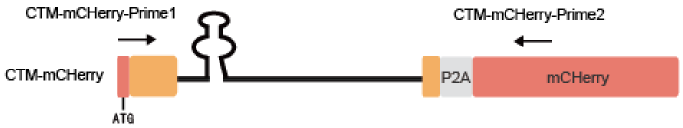

CTM-mCHerry-Prime1	CCACCATGGAAGACGCCAAA
CTM-mCHerry-Prime2	GATGGCCATGTTGTCCTCCTC

CTM-mCherry-Prime1 and CTM-mCherry-Prime2 were used for Figure 4A.

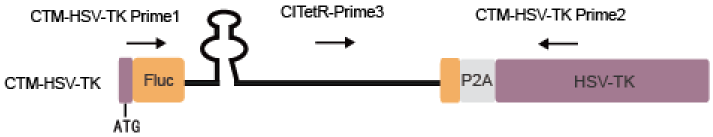

CTM-HSV-TK Prime1	CCACCATGGAAGACGCCAAA
CTM-HSV-TK Prime2	GACTTCCGTGGCTTCTTGCT
CITetR-Prime2	GCGTAAGTGATGTCCACCTCG

CTM-HSV-TK Prime1 and CTM-HSV-TK Prime2 were used for Supplementary Figure 5A, while CTM-HSV-TK Prime1 and CITetR-Prime2 were used for Supplementary Figure 5B.

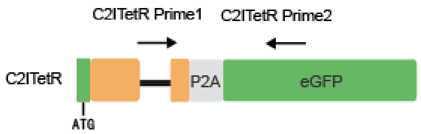

C2ITetR Prime1	AAGGGCGTAAGTATCCGGC
C2ITetR Prime2	GCTTCATGTGGTCGGGGTAG

C2ITetR Prime1 and C2ITetR Prime2 were used for Figures 8B and 9B.

Figure S9. Information on primers and templates
